# Molecular Characterization of the Von Willebrand Factor Type D Domain of Vitellogenin from *Takifugu flavidus*

**DOI:** 10.3390/md19040181

**Published:** 2021-03-25

**Authors:** Kun Qiao, Caiyun Jiang, Min Xu, Bei Chen, Wenhui Qiu, Yongchang Su, Hua Hao, Zhiyong Lin, Shuilin Cai, Jie Su, Zhiyu Liu, Wenshu Huang

**Affiliations:** 1 Key Laboratory of Cultivation and High-value Utilization of Marine Organisms in Fujian Province, Fisheries Research Institute of Fujian, Xiamen 361013, China; qiaokun@xmu.edu.cn (K.Q.); xumin1315@foxmail.com (M.X.); chenbeifjfri@foxmail.com (B.C.); suyongchang@126.com (Y.S.); allensky@63.com (S.C.); sjscut@126.com (J.S.); 2 Fisheries College, Jimei University, Xiamen 361021, China; jiangcaiyun626@163.com; 3 State Environmental Protection Key Laboratory of Integrated Surface Water-Groundwater Pollution Control, School of Environmental Science and Engineering, Southern University of Science and Technology, Shenzhen 518055, China; qiuwh@sustc.edu.cn; 4 State Key Laboratory of Marine Environmental Science, College of Oceanography and Environmental Science, Xiamen University, Xiamen 361005, China; hhao@xmu.edu.cn (H.H.); linzhiyong@xmu.edu.cn (Z.L.)

**Keywords:** *Takifugu flavidus*, tetrodotoxin, vitellogenin VWD domain, transport, toxin-binding protein

## Abstract

The von Willebrand factor type D (VWD) domain in vitellogenin has recently been found to bind tetrodotoxin. The way in which this protein domain associates with tetrodotoxin and participates in transporting tetrodotoxin in vivo remains unclear. A cDNA fragment of the vitellogenin gene containing the VWD domain from pufferfish (*Takifugu flavidus)* (TfVWD) was cloned. Using in silico structural and docking analyses of the predicted protein, we determined that key amino acids (namely, Val^115^, ASP^116^, Val^117^, and Lys^122^) in TfVWD mediate its binding to tetrodotoxin, which was supported by in vitro surface plasmon resonance analysis. Moreover, incubating recombinant rTfVWD together with tetrodotoxin attenuated its toxicity in vivo, further supporting protein–toxin binding and indicating associated toxicity-neutralizing effects. Finally, the expression profiling of TfVWD across different tissues and developmental stages indicated that its distribution patterns mirrored those of tetrodotoxin, suggesting that TfVWD may be involved in tetrodotoxin transport in pufferfish. For the first time, this study reveals the amino acids that mediate the binding of TfVWD to tetrodotoxin and provides a basis for further exploration of the molecular mechanisms underlying the enrichment and transfer of tetrodotoxin in pufferfish.

## 1. Introduction

Pufferfish is a fish belonging to the class Osteichthyes and the order Tetraodontiformes. China is the world’s largest producer of pufferfish, accounting for 88% of the total global aquaculture production. The main fugu breeding species include *Takifugu rubripes, T. obscurus, T. flavidus*, and *T. bimaculatus* [1]. The pufferfish has delicate meat and a delicious taste, but it also contains the deadly toxin tetrodotoxin (TTX), which often leads to food poisoning deaths from consumption by people. As a main commercial breeding species, the research on *T. flavidus* is currently focused on artificial breeding, feed nutrition, etc. [2]; toxicological studies involving TTX transport and enrichment in *T. flavidus* have not been reported. The completion of the genome mapping of *T. flavidus* provides favorable reference data for the in-depth exploration of functional genes and the verification and analysis of protein functions [3]. Recently, the von Willebrand factor type D (VWD) domain of vitellogenin was reported to bind TTX in *T. flavidus*. To the best of our knowledge, no investigation has been performed examining the function of the VWDs in *T. flavidus* and their relationship to TTX transport and enrichment.

TTX is a small-molecule alkaloid neurotoxin that functions as a typical Na^+^ channel blocker. It can specifically bind to the receptor of Na^+^ channels on the surface of muscle nerve cells and can inhibit the influx of Na^+^ in nerve cells, thus blocking excitation transmission between nerves and muscles [4]. Although first discovered in pufferfish, TTX has since been identified in several terrestrial and aquatic organisms, including *Taricha torosa, Colostethus inguinalis, Atergatis floridus,* and *Tutufa lissostoma* [5,6]. Notably, the tolerance of different organisms to TTX varies markedly, and pufferfish toxicity shows considerable individual and regional variations [7]. Toxic pufferfish that accumulate large amounts of TTX are endowed with a higher resistance to TTX than that of non-toxic organisms. Among the poisonous pufferfish, the liver and ovaries generally show the highest toxicity; however, the mechanism of TTX transfer and accumulation in pufferfish organs remains unclear. Feeding non-toxic cultured *Takifugu rubripes* or *T. niphobles* food containing TTX results in a rapid accumulation of toxin in their livers and ovaries [8,9]. In comparison, intramuscular injection of TTX into four-month-old *T. rubripes* led to TTX being first transferred to the liver through the blood and then enriched in the skin [10]. However, a significant difference in TTX enrichment was observed between male and female pufferfish, and TTX concentration in the ovaries was significantly higher than that in the testes [11,12,13].

TTX-binding proteins play an essential role in the transfer and enrichment of TTX in vivo. Shiomi et al. [14] first identified high molecular weight substances in shore crabs (*Hemigrapsus sanguineus*) that displayed neutralizing effects against the lethal activity of TTX. Matsui et al. [15] isolated and purified a TTX-binding protein from the plasma of the pufferfish *T. niphobles* and tested its binding ability for TTX. The binding reversibility between the TTX-binding protein and TTX suggested that the protein plays a role in TTX transfer and transport in pufferfish [15]. Subsequently, a pufferfish saxitoxin- and tetrodotoxin-binding protein (PSTBP) was purified from the plasma of *T. pardalis*, consisting of noncovalently linked dimers of a single subunit; two highly homologous cDNAs encoding pufferfish PSTBP1 and PSTBP2 were subsequently obtained [16]. Moreover, high molecular weight TTX-binding substances were isolated from the muscle of five toxic gastropods, with the molecular weight of the TTX-binding protein of *Natica lineata* estimated at approximately 434 kDa [17].

Yin et al. [18] purified a toxin-binding protein from the ovaries of *T. pardalis* by ion-exchange high-performance liquid chromatography. The 10 kDa purified protein (TPOBP-10) was homologous to the predicted vitellogenic von Willebrand factor (vWF) type D (VWD) domain of *T. rubripes*. Vitellogenin generally contains three conserved domains: the lipoprotein amino-terminal region located at the N-terminus, the DUF 1943 of unknown function, and the VWD domain at the C-terminus. The VWD domain has been found in the vitellogenin protein of some vertebrates, crustaceans, and insects [19,20]. The VWD domain elicits the binding of oocyte membrane receptors to vitellogenin [21], resulting in vitellogenin uptake into oocytes. Furthermore, VWDs are present in several other proteins, such as mucins and zonadhesins, which promote the binding of vitellogenin to its receptor. The sphericity of this domain enhances its lubricity in mucin [22] and underlies the adhesion function in other proteins such as integrins and zonadhesins [23]. The manner in which this protein binds TTX and participates in TTX transport in pufferfish remains unclear. Further study of the transport and organ enrichment of TTX in *T. flavidus* will provide a theoretical basis and reference for the development of effective measures to reduce or block the production of TTX. This, in turn, should reduce the risk of eating pufferfish and provide a guarantee for its safe consumption.

Herein, we hypothesized that the VWD domain of vitellogenin can bind TTX and mediate TTX transport in *T. flavidus*. To test this hypothesis, we cloned a cDNA fragment containing the VWD domain from pufferfish (*Takifugu flavidus*) (TfVWD) domain of the vitellogenin gene from the liver of *T. flavidus*. We then studied the binding activity of TfVWD by performing site-specific mutagenesis of the recombinant expression product at key sites. The purified recombinant expression products rTfVWD and rTfVWD^mut^ were obtained. The TTX-binding abilities of the recombinantly expressed products were analyzed using surface plasmon resonance (SPR). Furthermore, the distribution of TfVWD transcripts and proteins was evaluated using quantitative polymerase chain reaction (qPCR) and Western blotting, respectively, in different tissues and developmental stages.

## 2. Results

### 2.1. Molecular Characteristics of the TfVWD Domain in T. flavidus

**Figure 1:**
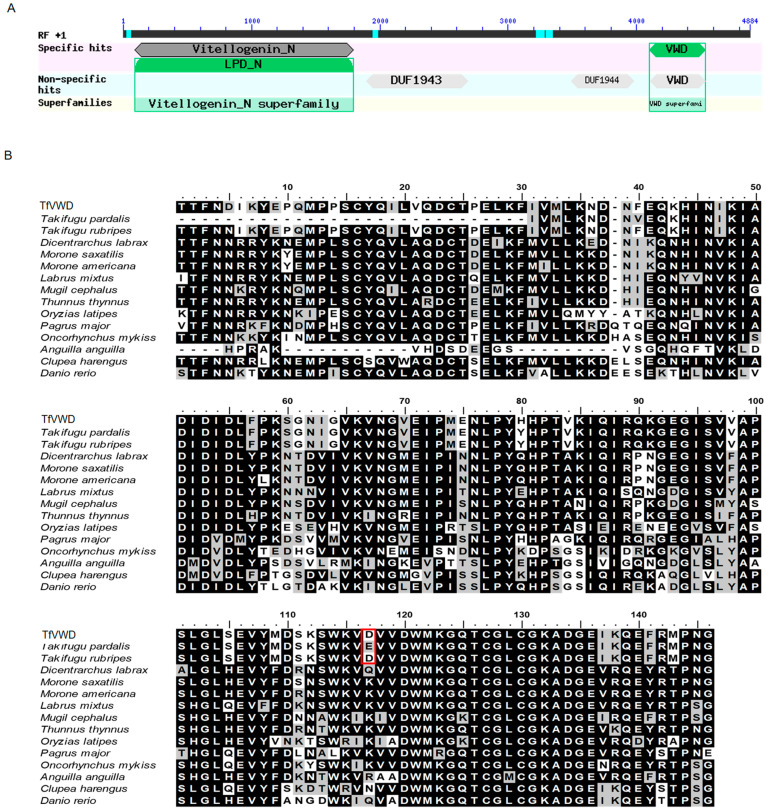
TfVWD gene sequence analysis and domain prediction. (**A**) Structural composition of the vitellogenin gene. (**B**) Alignment of the VWD domains in *Takifugu flavidus* vitellogenin with those from other fish species. Multiple sequence alignment of TfVWD (MT160192) and *T. pardalis* (A0A292G9J6), *T. rubripes* (LOC101072500), *Dicentrarchus labrax* (AFA26671.1), *Morone saxatilis* (ADZ57174.1), *Morone americana* (AAZ17417.1), *Labrus mixtus* (ACK36969.1), *Mugil cephalus* (BAF64837.1), *Thunnus thynnus* (ADD63988.1), *Oryzias latipes* (XP_004067788.1), *Pagrus major* (BAE43872.1), *Oncorhynchus mykiss* (BBA57869.1), *Anguilla* (Q3ZUB9), *Clupea harengus* (ACJ65210.1), and *Danio rerio* (LOC110438452). Identical amino acids are shaded in black, and similar amino acids are shaded in gray. The numbers on the top show the amino acid’s position within the corresponding species. Asp^116^ in *Takifugu flavidus* and *T. rubripes* and Glu^86^ in *T. pardalis* are shown boxed in red. VWD, von Willebrand factor type D; TfVWD, VWD from *Takifugu flavidus*.

The 4884 bp cDNA fragment of the vitellogenin gene was cloned from the liver of *T. flavidus* (GenBank: MW603776; Figure S1). The conserved domains were predicted using the NCBI website (https://www.ncbi.nlm.nih.gov/Structure/cdd/wrpsb.cgi (accessed on 18 February 2021)), revealing that the cloned fragment was comprised of vitellogenin_N, DUF1943, DUF1944, and VWD domains (Figure 1A). The VWD sequence (underlined in Figure S1) was cloned by designing specific primers (shown in bold) and temporarily designated as TfVWD (GenBank: MT160192). TfVWD encodes 145 amino acids, generating a polypeptide with a molecular weight of 16.49 kDa and a predicted isoelectric point of 5.59. In the predicted TfVWD domain, the first conserved Cys appears to form a disulfide bridge with the second conserved Cys (shaded in gray in Figure S1). The multi-sequence alignment of the VWDs in *T. flavidus* vitellogenin with that of other fish species is shown in Figure 1B. The percent amino acid identity between the *T. flavidus* and *T. rubripes* sequences was 98.62%. The VWD sequences of other fishes were obtained from GenBank.

### 2.2. Protein Modeling and Molecular Docking

**Figure 2:**
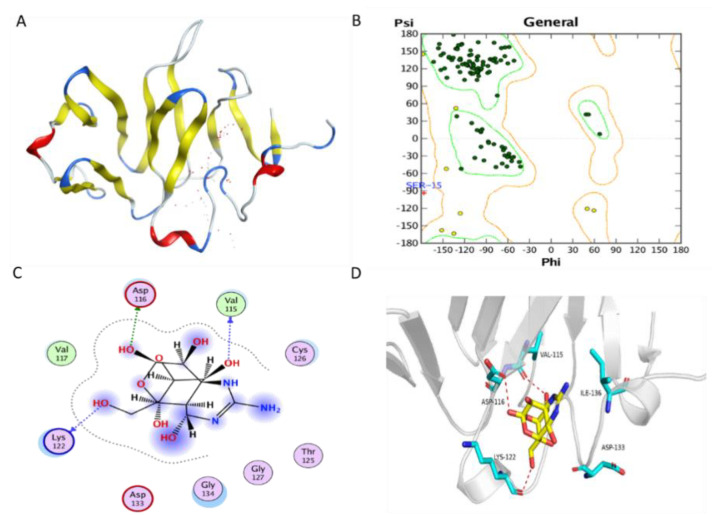
Protein modeling of TfVWD and molecular docking of TfVWD and TTX. (**A**) Model of TfVWD predicted using ROBETTA and QUARK. The figure shows the predicted binding site in dots in white and red, and depicts the protein in ribbon. Helix, turn, and beta-sheet are colored in red, blue, and yellow, respectively. (**B**) Ramachandran plot for TfVWD. Dark green dots represent the residues in the favored regions, yellow dots represent the residues in the allowed regions, and the red cross represents the residues in the irrational regions. (**C**) Binding model depicted in 2D. Green dotted arrows represent hydrogen bonding with the sidechain, and blue arrows indicate hydrogen bonding with the backbone. (**D**) Binding model depicted in 3D. Ligands are colored yellow and the surrounding residues in the binding pocket are colored cyan. The backbone of the receptor is depicted as a light gray ribbon. The red dotted lines show the hydrogen bonds between the ligand and the receptor. The surface view depicts the receptor as white and the ligand as yellow. TfVWD, von Willebrand factor type D from *Takifugu flavidus*; TTX, tetrodotoxin.

The TfVWD model was ab initio predicted using ROBETTA [24] and QUARK [25], and its structure is shown in Figure 2A. The modeled TfVWD protein, its potential binding sites, and the Ramachandran plot for the best-modeled structure, generated using ROBETTA, are shown in Figure 2B. Almost all amino acid residues were located in the favored or allowed regions, with only one outlier, which indicates that the modeled 3D structure is feasible.

The docking (binding energy) score of TTX to the ab initio predicted protein was −4.872 kcal/mol. The binding sites were chosen by AutoDock Vina. The binding mode is illustrated in both 2D and 3D (Figure 2C,D, respectively). The putative binding pocket consisted of Ser^15^, Cys^16^, Tyr^17^, Gln^18^, Ser^112^, Trp^113^, Lys^114^, Val^115^, Asp^116^, Val^117^, Met^121^, Lys^122^, Gly^123^, Gln^124^, Thr^125^, Cys^126^, Gly^127^, Leu^128^, Lys^131^, Ala^132^, Asp^133^, Gly^134^, Glu^135^, Ile^136^, Glu^139^, and Phe^140^. One hydroxyl oxygen atom forms a hydrogen bond with the oxygen atom in the sidechain of Asp^116^; another two hydroxyl oxygen atoms form hydrogen bonds with oxygen atoms in the backbone of Val^115^ and Lys^122^ in the receptor. The ligand afforded favorable van der Waals interactions with Val^117^ in this receptor.

### 2.3. Recombinant Expression of TfVWD

**Figure 3:**
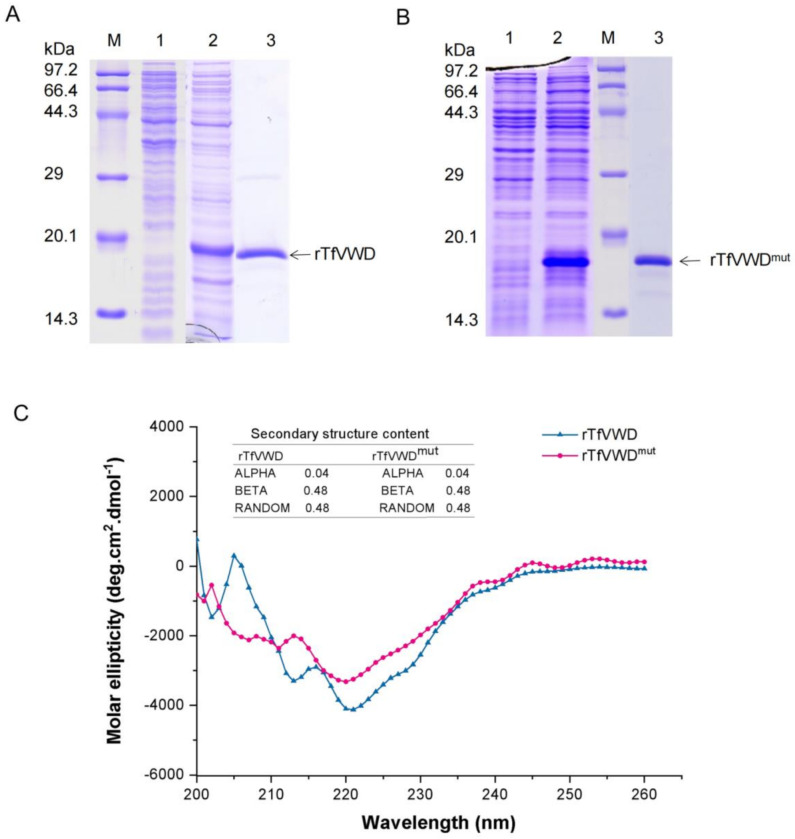
Recombinant expression and purification of rTfVWD and rTfVWD^mut^. (**A**) SDS-PAGE analysis of the purified rTfVWD protein. Lane M: Premixed Protein Marker (TaKaRa); lane 1: Proteins prior to IPTG induction; lane 2: Proteins following IPTG induction; lane 3: Purified rTfVWD protein. (**B**) SDS-PAGE analysis of the purified rTfVWD^mut^ protein. Lane M: Premixed Protein Marker; lane 1: Proteins prior to IPTG induction; lane 2: Proteins following IPTG induction; lane 3: Purified rTfVWD^mut^ protein. TfVWD, von Willebrand factor type D from *Takifugu flavidus*; TTX, tetrodotoxin; rTfVWD, recombinant TfVWD protein; rTfVWD^mut^, recombinant TfVWD mutated protein. (**C**) Secondary structure of rTfVWD and rTfVWD^mut^ evaluated by circular dichroism.

To further investigate the in vitro activity of the protein, TfVWD was expressed recombinantly in *Escherichia coli*. A 435 bp PCR product encoding 145 amino acids was cloned into the pET-28a expression vector (Figure S2). The expressed TfVWD product contained 20 amino acid residues in the N-terminal 6 × His-Tag, and had a theoretical molecular weight and isoelectric point of 18.64 kDa and 6.54, respectively. Protein expression was induced using IPTG; the expressed rTfVWD products existed in the form of inclusion bodies and soluble supernatant, with most of the protein located in the supernatant. The optimal expression conditions were determined to be incubation with an initial cell concentration of OD600 = 0.2 at 37 °C for 6 h, resulting in a stably expressed product with a molecular weight of approximately 18 kDa (Figure 3A). The recombinant expression of rTfVWD^mut^, using the same optimized conditions, is shown in Figure 3B. Immobilized metal–chelate affinity chromatography using nickel ions was used for recombinant protein purification. The protein-containing cell supernatant was collected following ultrasound treatment; rTfVWD and rTfVWD^mut^ specifically bound nickel ions in the affinity chromatography column (Figure 3A,B). We further compared the secondary structures of rTfVWD and rTfVWD^mut^ by circular dichroism, and the results in Figure 3C show that the two proteins contain the same content of secondary structures.

### 2.4. SPR Measurement of the Binding Activity of TTX and Recombinant Proteins

**Figure 4:**
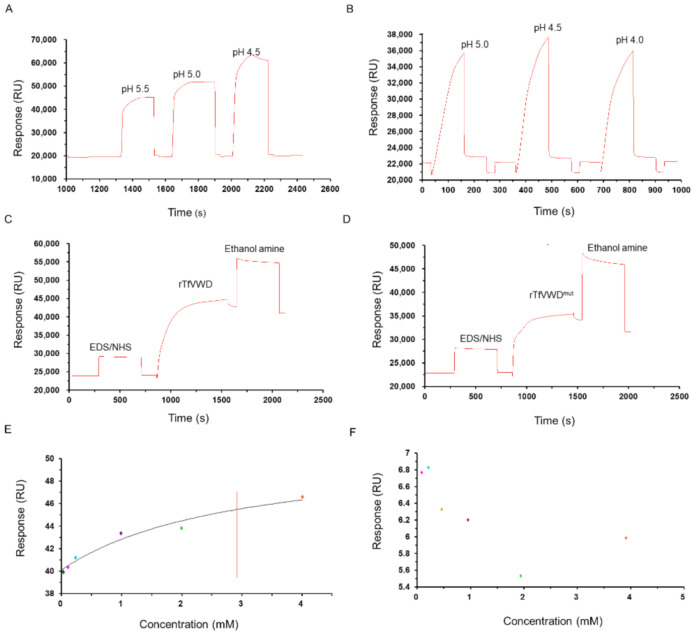
Binding activity of rTfVWD and rTfVWD^mut^ with TTX. Screening of the optimal coupling pH of rTfVWD (**A**) and rTfVWD^mut^ (**B**) on the surface of the CM5 chip. Response diagram of CM5 chip surface binding with rTfVWD (**C**) or rTfVWD^mut^ (**D**). Steady-state fitting to obtain the K_D_ of rTfVWD (**E**) and rTfVWD^mut^ (**F**). The red line shows the K_D_ of rTfVWD. TfVWD, von Willebrand factor type D from *Takifugu flavidus*; TTX, tetrodotoxin; rTfVWD, recombinant TfVWD protein.

Recombinant proteins were diluted with sodium acetate buffer with pH values of either 5.0, 4.5, or 4.0. After the sample was injected, the pH value with the largest coupling amount (pH 4.5; Figure 4A,B) was selected for subsequent experiments. The final coupling amounts of rTfVWD and rTfVWD^mut^ on the CM5 chip surface were 18,239.3 and 10,992.1 RU (Figure 4C,D). TTX crude products at different concentrations were flowed through the chip with affixed rTfVWD and rTfVWD^mut^ to measure the interaction between the ligand and receptor. The results showed that the response value (Rmax) of combining rTfVWD and TTX was 46.5 RU, and the equilibrium dissociation constant (K_D_) of rTfVWD and TTX was calculated using steady-state fitting (affinity) to be 2.9 × 10^−3^ M (Figure 4E). However, rTfVWD^mut^ did not bind TTX (Figure 4F).

### 2.5. In Vivo Effects of rTfVWD on Mice

**Figure 5:**
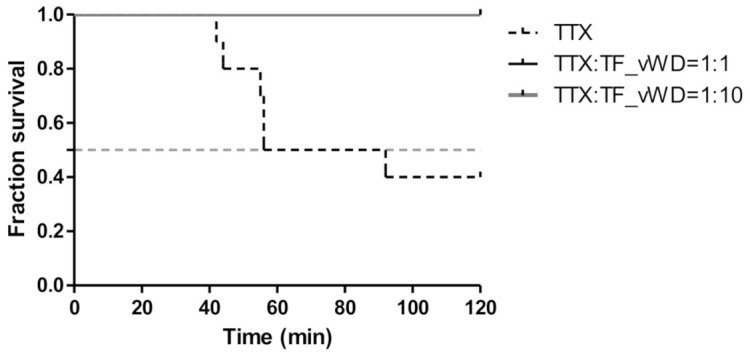
Survivorship curve of the mice administered a mixture of TTX and rTfVWD by gavage. TfVWD, von Willebrand factor type D from *T. flavidus*; TTX, tetrodotoxin; rTfVWD, recombinant TfVWD protein.

As shown in Figure 5, all the mice survived the gavage administration of a mixture of TTX and protein, and no clinical symptoms of toxicity were observed during the initial 2 h, suggesting that rTfVWD can neutralize TTX toxicity.

### 2.6. Distribution of the TfVWD Domain in the Tissues of T. flavidus

**Figure 6:**
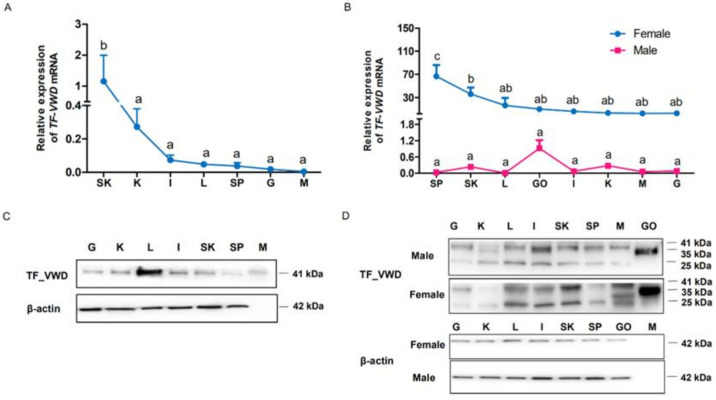
Tissue distribution of TfVWD mRNA and protein in cultured *Takifugu flavidus* at different developmental stages. (**A**) TfVWD transcript levels in different tissues of immature *T. flavidus* determined by qPCR. SK, skin; K, kidneys; I, intestines; L, liver; SP, spleen; G, gills; M, muscle. (**B**) TfVWD transcript levels in the different tissues of mature male and female *T. flavidus* determined by qPCR. SP, spleen; SK, skin; L, liver; GO, gonads; I, intestines; K, kidneys; M, muscle; G, gills. Significant differences between the different tissues (*p* < 0.05; *n* = 6) in panels A and B were calculated using one-way ANOVA followed by Tukey’s test and denoted by different letters (a–d). (**C**) Western blot showing TfVWD protein tissue distribution in immature *T. flavidus*. G, gills; K, kidneys; L, liver; I, intestines; SK, skin; SP, spleen; M, muscle. (**D**) Western blot of TfVWD protein tissue distribution in mature male and female *T. flavidus*. G, gills; K, kidneys; L, liver; I, intestines; SK, skin; SP, spleen; M, muscle; GO, gonads. ANOVA, analysis of variance; TfVWD, von Willebrand factor type D from *T. flavidus*; TTX, tetrodotoxin.

To investigate the expression of TfVWD at the transcript and protein levels among various tissues and different developmental stages of *T. flavidus*, qPCR and Western blotting were performed, respectively. In immature *T. flavidus*, the level of TfVWD mRNA in the skin was significantly higher than that in other tissues (*p* < 0.05, Figure 6A). In mature female pufferfish, the highest levels of TfVWD mRNA were found in the spleen and skin, being significantly higher than that in other tissues (*p* < 0.05, Figure 6B). In the mature male pufferfish, the highest expression level was observed in the testes.

Western blot analysis showed a VWD protein band at 41 kDa in the immature pufferfish, which was mainly distributed in the liver, followed by the skin, spleen, and kidneys (Figure 6C). In contrast, different bands of TfVWD were detected in adult pufferfish, with positive results obtained from the soluble protein extracts of all tissues (Figure 6D). A 35 kDa band was detected in the gonads, whereas 41 kDa and 25 kDa bands were detected in other tissues. In females, the highest level of TfVWD protein was observed in the ovaries, followed by the skin and liver; however, in males, the highest expression levels were in the testes, followed by the intestines, with the lowest expression in the kidneys (Figure 6D).

## 3. Discussion

In this study, we cloned the vitellogenin gene of *T. flavidus*, and confirmed the molecular structure of the VWD. In agreement with the finding of Yin [18], our multiple sequence alignment results showed that this domain is evolutionarily conserved in fish, and consists of a β′ component (β′-c) and a C-terminal coding region. This domain is cysteine-rich and constitutes a highly structured region that plays an essential role in oligomer polymerization through disulfide bond formation [26]. By comparing differences in some of the amino acid residues in the VWD domain in other fish that do not carry TTX, some important amino acids were identified [18].

When constructing the 3D structure of TfVWD, we first evaluated the feasibility of homology modeling before using the de novo model. The VWD protein sequence was analyzed by BLAST on the NCBI website, and the crystal structure of chain B lipovitellin (1LSH), with the highest homology of only 36%, was obtained by comparison. This crystal structure has not been resolved to the VWD. Thus, this crystal structure cannot be used as a template for homology modeling. Therefore, the three-dimensional structure of the sequence was predicted from ab initio calculations. Through the method of molecular docking, we identified the putative binding pocket where TfVWD binds to TTX, consisting of two α-helices with two turns and one β-sheet. The key protein binding sites for TTX were obtained for the first time, which provides useful information for in-depth study of the interactive role of proteins and toxins.

The key sites for rTfVWD binding with TTX were determined by site-directed mutagenesis. Using the Biacore T200 biomolecule interaction analysis system, rTfVWD was set as the ligand and TTX, as the analyte to analyze the affinity of the interaction. According to experimental data, affinity can usually be evaluated through kinetic constant calculation and steady-state methods. In this study, our experimental sensorgrams were of the “rapid rise and fall” type (rapid combination and rapid dissociation), which is suitable for steady-state model analysis. That is, in the equilibrium state, the affinity is obtained by directly fitting a graph of the amount of bound complex to the analyte concentration (Figure 4E,F). The protein–small molecule binding affinity constant or K_D_ value is usually in the range of 10^–3^ to 10^–6^ M. Our results showed that the rTfVWD protein could bind TTX with an equilibrium dissociation constant of 2.92 × 10^−3^ M, which is considered weak affinity. This suggests that the VWD peptide of vitellogenin may assist in transferring TTX to other tissues and organs [27]. The mutant recombinant protein failed to bind TTX, verifying the molecular docking results, i.e., that key hydrogen bonds formed by Asp^116^, Val^115^, Lys^122^, and Val^117^, and van der Waals forces formed. Notably, based on the analysis of amino acid sequence differences of the VWD in different fishes and docking with TTX, Asp^116^ in the VWD is substituted in other fish species that do not generate TTX (Figure 1B). As a result, the substituted amino acids are unable to form hydrogen bonds with the hydroxyl oxygen atoms on TTX. This finding suggests that Asp^116^ is the key amino acid mediating VWD binding to the toxin.

This study confirmed the TTX binding activity of recombinant protein rTfVWD. Previous studies have shown that the VWD recombinant protein can combine with lipoteichoic acid (LTA) and lipopolysaccharides (LPS) to promote the phagocytic activity of macrophages by serving as a pattern recognition receptor stimulating immunological functions [28]. We found that rTfVWD and TTX exhibited a certain binding ability in vitro. This conclusion supports the previous hypothesis that the VWD, a fragment of the vitellin pro-peptide found in the ovaries of mature pufferfish, can specifically bind TTX [18]. Therefore, we evaluated the neutralizing effect of the rTfVWD protein on TTX toxicity in vivo using gavage administration in mice. Notably, the survival time of mice receiving a previously co-incubated combination of rTfVWD and TTX was significantly prolonged, and the mortality rate was significantly reduced compared to that following TTX administration alone. Thus, the toxicity of TTX was reduced through co-incubation with rTfVWD, which is similar to the findings of puffer binding protein from shore crabs and five gastropods [14,17]. This indicates that, although TTX is toxic alone, it is non-toxic in the combined state.

To better understand the role of the VWD in vivo, the distribution of mRNA expression and protein content of TfVWD *T. flavidus* was evaluated. Yin et al. [18] showed that TPOBP-10 mRNA was most highly expressed in the ovaries and liver of adult female *T. pardalis*. However, our findings of high expression in the spleen and skin in *T. flavidus* differ from that of previous research. This difference is mainly due to the different species of pufferfish and the different methods of gene quantification.

The Western blot results showed that various forms of VWD protein bands appeared in different developmental stages and different tissues and organs. A complete vitellogenin protein comprises a signal polypeptide, a heavy chain lipovitellin, phosvitin, light chain lipovitellin, a VWD containing a β′component (β′-c), and a C-terminal coding region. Vitellogenins are acidified by the action of proton pumps and cathepsin D, and consequently, they can be activated and cleaved into their individual constituents [29]. Therefore, the different band sizes detected by Western blotting may be due to the combination of different domains produced by the variable cleavage of the protein. In addition, we also found that the tissues and organs of juvenile fish showed a single band, whereas multiple bands were detected in samples taken from adult fish. Vitellogenin is synthesized in the liver under the influence of gonadotropin (GtH)-induced ovarian estradiol-17β (E2), and transported through the blood to the ovaries, where it is absorbed and processed by oocytes into its derivative yolk protein [29]. Therefore, the composition of vitellogenin is different in various tissues and organs; hence, regarding the presence of different banding patterns, the Western blots showed that the band patterns in the different tissues were different, which is to be expected.

The Western blot results also showed that VWD protein expression in the liver was the highest in immature pufferfish. In mature fish, the VWD protein content in the ovaries and testes was highest in female and male fish, respectively. These results are consistent with those of previous studies. For example, Li et al. [30] expressed the VWD of *Eriocheir sinensis* and prepared an antibody for it, revealing the highest expression in ovarian tissue, followed by hepatopancreas and hemolymph, by Western blotting. Notably, the expression characteristics of the VWD were consistent with the tissue distribution of TTX in pufferfish, which exhibited the highest accumulation in the liver and ovaries, followed by the intestines and skin. Female pufferfish transfer accumulated TTX to the ovaries during the spawning period, which can transmit toxins to larvae through ovulation, thereby helping pufferfish larvae and juveniles to protect themselves from external predators [7,11]. It has been speculated that this is attributed to the large accumulation of TTX in pufferfish ovaries [31,32]. Although the male puffer releases TTX from the skin, most TTX remains in the liver and skin [33]. Vitellogenin is primarily synthesized in the liver and is distributed throughout the body through systemic circulation and carried to the ovaries [34]. In addition to its role as a yolk protein precursor, yolk proteinogen acts as a carrier protein for metals, inorganic phosphates, lipids, and carbohydrates [35]. In this study, we found that rTF–VWD can be combined, though weakly and reversibly, with TTX. Therefore, we speculate that the VWD of vitellogenin plays a role in TTX transport in pufferfish. Nevertheless, further investigation is needed to fully unravel how the VWD transports TTX in vivo and which transcription factors are involved in regulation.

## 4. Materials and Methods

### 4.1. Experimental Animals and Tissue Collection

*Takifugu flavidus* was purchased from a local aquaculture farm in ZhangPu County, Fujian Province, China. Immature and mature male and female fish were individually collected. Next, 0.01% eugenol cement was used to anesthetize the pufferfish before dissection. The gill, liver, stomach, intestine, kidney, heart, muscle, skin, spleen, and gonad tissues were individually collected for RNA extraction. Six fish were collected for each group. The samples were rapidly placed in liquid nitrogen and stored at −80 °C.

### 4.2. Total RNA Extraction and Cloning of the TfVWD Gene

The total RNA from the liver of *T. flavidus* was extracted according to the manufacturer’s instructions using the RNAprep pure Tissue Kit (DP431, Tiangen, Beijing, China). The concentration and purity of the total RNA solution were determined using an Infinite200Pro microplate reader (TECAN, Männedorf, Switzerland), and reverse transcription was carried out according to the operating instructions of the TransScript One-Step gDNA Removal and cDNA Synthesis SuperMix (Transgen Biotech, Beijing, China).

The complete coding sequence (GenBank: MW603776) of the vitellogenin gene of *T. flavidus* was obtained by transcriptome sequencing. A pair of primers (VWD Forward-[F]; 5′-CTA CGC CCA GAA CTT GCT GAC-3′; VWD-Reverse [R]: 5′-GCA CTC GCT GTT GTC TCT GC-3′) was designed, targeting the open reading frame of TfVWD using Primer Premier 5.0. PCR amplification was performed using liver cDNA and the following reaction conditions: initial denaturation at 95 °C for 3 min; 32 cycles of denaturation at 95 °C for 30 s, annealing at 60 °C for 30 s, and elongation at 72 °C for 90 s; the final cycle was followed by an extension at 72 °C for 10 min. The PCR products were run on an agarose gel, purified using a gel extraction kit (Axygen, Union City, CA, USA), and then cloned into vector pMD19-T using a TA cloning kit (TaKaRa Bio., Inc., Shiga, Japan) for sequencing by Bioray (Shanghai, China).

### 4.3. Construction and Identification of a Recombinant Expression Vector

The PCR product was ligated with pMD19-T to obtain a recombinant-positive plasmid containing the TfVWD target gene. This positive plasmid was used as an amplification template. The following primers, specific to the *T. flavidus* TfVWD gene, were designed based on the multiple cloning sites of the pET-28a (Novagen) vector: Upstream primer F1, 5′-GGA ATT CCA TAT GAC CAC CTT CAA CGA CAT CAA-3′ and downstream primer R1, 5′-CCG CTC GAG TCA CCC GTT GGG CAT ACG-3′. The underlined sequences represent the endonuclease sites for *Nde*I and *Xho*I (TaKaRa Bio. Inc., Shiga, Japan), respectively. TfVWD F1 and TfVWD R1 were used to amplify the target gene sequence using TransStart FastPfu high-fidelity DNA polymerase (TransGen Biotech Co., Ltd., Beijing, China). The PCR product and the expression plasmid were digested with the corresponding restriction enzymes and ligated using T4 DNA ligase (TaKaRa Bio. Inc. Shiga, Japan). The recombinant expression plasmid was confirmed by PCR and DNA sequencing.

Correspondingly, the mutant vector plasmid pET-28a/TfVWD^mut^ was constructed according to the predicted results of TfVWD protein docking with TTX (see below). All predicted amino acids, i.e., Asp^116^, Val^115^, and Lys^122^, were mutated to alanine (the corresponding codon was mutated to “gcg”). *Nde*I and *Xho*I restriction sites were added to the two ends, and mutant plasmid synthesis was commissioned through Sangon Biotech Co., Ltd. (Shanghai, China).

### 4.4. Expression and Purification of Recombinant TfVWD

A single colony of *Escherichia coli* BL21 (DE3) pLysS (Vazyme, Nanjing, China) transformed with pET-28a/TfVWD and pET-28a/TfVWD^mut^ was inoculated into LB medium containing 50 mg/mL of kanamycin, cultured at 37 °C, until the optical density at 600 nm (OD_600_) reached 0.2–0.5. Protein expression in the cells was then induced by adding isopropyl β-d-1-thiogalactopyranoside (IPTG) to a final concentration of 0.2 mM and incubated at 37 °C for 6 h. The harvested cells were frozen, thawed, and sonicated to separate the soluble and insoluble portions, which were collected and analyzed by 12% (*w*/*v*) SDS-PAGE.

The purification procedure was carried out using an AKTA Purifier 100 (GE Healthcare Life Sciences, Waukesha, WI, USA) and immobilized metal affinity chromatography. The collected supernatants containing the soluble component of rTfVWD and rTfVWD^mut^ were filtered using a 0.45 mm filter membrane. We utilized MilliQ water to wash the prepacked column (HisTrapTM FF Crude 5 mL, GE Healthcare Life Sciences), after which 3–5 column volumes of equilibration buffer (10 mM PBS + 40 mM imidazole, pH 7.4) were used to equilibrate the column. The elution peaks of the protein were collected using elution buffer (10 mM PBS + 400 mM imidazole, pH 7.4), and small amounts were validated using SDS-PAGE. The purified protein was subjected to SDS-PAGE, and the target band was excised for matrix-assisted laser desorption/ionization time-of-flight/time-of-flight mass spectrometry (MALDI-TOF/TOF-MS) protein mass spectrometry analysis.

### 4.5. CD Spectroscopy

In order to investigate the secondary structure of rTfVWD and rTfVWD^mut^, the recombinant proteins were solubilized in PBS (pH 7.4) and the protein concentration was adjusted to 25 μM/L. Samples were scanned with a circular dichroism chromatograph using a quartz cuvette with a 1.0 mm path length, a scanning light range of 200–260 nm with a 2.0 nm spectral bandwidth, and a scanning speed 0.1 s/nm. The obtained CD spectra were then converted to the average residue ellipticity as described by Chou et al. [36], with the following equation:

θ_M_ = (θ_obs_ × 1000)/(c × l × n)

where θ_M_ is the molar ellipticity ((deg × cm^2^)/dmol), θ_obs_ is the observed ellipticity corrected for the buffer at a given wavelength (mdeg), c is the peptide concentration (mM), l is the path length (mm), and n is the number of amino acids.

### 4.6. Protein Modeling and Molecular Docking

Lacking a suitable template for the homology modeling of the TfVWD protein subdomain, the ab initio prediction of TfVWD was conducted using ROBETTA [24] and QUARK [25], and the top five results were downloaded and analyzed. The quality of the predicted model was evaluated by calculating the Phi-Psi angles (i.e., Ramachandran plot), which was conducted using the Protein Geometry module in MOE, version 2014.09 (Chemical Computing Group Inc., Montreal, QC, Canada).

MOE-dock was used for molecular docking simulations of the TTX ligand and for predicting its binding affinity with the TfVWD protein. The 2D structure of the TTX ligand was downloaded from PubChem (https://pubchem.ncbi.nlm.nih.gov/ (accessed on 10 August 2019), PubChem CID: 6324668) and converted to 3D in MOE through energy minimization. Prior to docking, the force field of AMBER10:EHT and the implicit solvation model of the reaction field (R-field) were selected. The putative binding sites were predicted using the Site Finder module. The docking workflow followed the “induced fit” protocol in which the side chains of the receptor pocket could move according to ligand conformations, with a constraint on their positions. The weight used for tethering side-chain atoms to their original positions was 10. All docked poses of molecules were ranked first according to London dG scoring. A force field refinement was then carried out on the top 30 poses, followed by a rescoring of generalized-Born volume integral/weighted surface area (GBVI/WSA dG). The docking results were prepared using PyMOL (DeLano Scientific LLC, San Francisco, CA, USA).

Molecular docking of TTX was also conducted using AutoDock Vina [37] through the use of the predicted TfVWD protein and by setting the whole TfVWD protein in the search space. Parameter num_modes and energy_range were set to 30 and 5, respectively. This extra docking procedure represented a supplementary means to predict binding sites.

### 4.7. SPR Measurement of the Binding Activity of TTX and Recombinant Proteins

SPR was performed using a Biacore T200 system (GE Healthcare Life Sciences) to analyze TTX-to-protein interactions. To monitor the interactions of TTX with TfVWD protein, rTfVWD and rTfVWD^mut^ were immobilized on a CM5 Sensor Chip. Before fixing the recombinant proteins on the sensor chip, the appropriate binding buffer pH was screened using PBS buffer as the working buffer, and the recombinant proteins were diluted with 10 mM sodium acetate buffer (pH 5.0, 4.5, and 4.0) to a final concentration of 30 μg/mL. The injection time was 120 s and the flow rate was 10 µL/min. The pH value with the largest coupling amount was selected for subsequent experiments, and the surface was regenerated using 50 mM NaOH for 30 s following each injection.

A 1:1 solution of 1-(3-dimethylaminopropyl)-3-ethyl carbodiimide hydrochloride (EDC) and N-hydroxysuccinimide (NHS) was prepared, and the surface of the sensor chip was activated at a flow rate of 10 µL/min and an injection time of 420 s. Subsequently, 30 μg/mL of recombinant protein solution was injected (prepared in pH 4.5 sodium acetate buffer; pH conditions as determined through the preliminary screening). Finally, 1 mol/L of ethanolamine hydrochloric acid-blocking solution was injected for 800 s to seal the activated chip surface.

The TTX standard was diluted to 0, 125, 250, 500, 1000, 2000, and 4000 µM in PBS buffer, placed in the rack according to the operation requirements, subjected to an injection time of 180 s and dissociation time of 300 s, and then transferred to the sample rack. Measurements were performed on the sensor chip, followed by elution with glycine at pH 2.0 after each measurement (flow rate 30 µL/min, time 30 s). The experiment was performed at 25 °C. The equilibrium dissociation constant (K_D_) of recombinant proteins and TTX was calculated from the binding and dissociation curves using the Biacore Evaluation Software (T200 Version 1.0).

### 4.8. Mouse Gavage Experiment

All animals were purchased from the Laboratory Animal Center of Xiamen University (no. XMULAC20211001), where the experiments were conducted. Healthy, specific pathogen-free male Institute of Cancer Research (ICR) mice (19.0–21.0 g) were selected and weighed. The animals were randomly divided into three groups of 10 animals each. PBS buffer was used to dilute crude TTX (Taizhou Kangte Biological Engineering Co., Ltd., Taizhou, China). For each control group, 0.5 mL of 7 µg/mL TTX solution was delivered by gavage. For the experimental groups, the mice were treated with TTX plus rTfVWD at 1:1 and 1:10 ratios, respectively. Following advance incubation of TTX with rTfVWD of the corresponding molar ratio at 15 °C for 15 h, 0.5 mL of the appropriate mixture was administered to each mouse by gavage. The survival time and the number of deaths within 2 h were recorded.

### 4.9. qPCR

qPCR reactions comprised a volume of 20 µL and included 8 ng of total RNA, 10 pmol of the specific primers qTfVWD-F (5′-TCG CTG ACA TCG ACA TCG ACC-3′) and qTfVWD*-*R (5′-CAG TCC ACA TCA ACC TTC CAT G-3′), and 10 µL of FastStart Universal SYBR Green Master (Rox) (Roche, Roswell, GA, USA). The reaction conditions were as follows: Preincubation at 95 °C for 600 s, followed by 45 cycles of denaturation at 95 °C for 10 s, annealing at 60 °C for 10 s, and extension at 72 °C for 10 s. The EF1-α primers (GenBank accession no. MT160192, qEF1-α-F: 5′-ACT GAG GTG AAG TCT GTG GAA ATG C-3′; qEF1-α-R: 5′-TTT GGT GGG TCG TTC TTG CTG TC-3′) were designed to amplify the internal reference gene (EF1-α). qPCR data were calculated using the 2^−∆∆CT^ method. Melt curve analysis was performed to analyze the specificity of the PCR products.

### 4.10. Preparation of a Polyclonal Antibody against rTfVWD

The recombinant protein rTfVWD was purified for antibody preparation. Ten BALB/C mice were used to produce an antibody against rTfVWD. The mice were purchased from the Laboratory Animal Center of Xiamen University (no. XMULAC20211001), where they were maintained for more than one month until the serum antibody titer reached the experiment requirements. Anti- rTfVWD IgG was purified using NAb™ Spin Kits (Thermo, Waltham, MA, USA). The concentration, titer, purity, and specificity of the purified anti-rTfVWD antibody were analyzed using an ultraviolet spectrophotometer, enzyme-linked immunosorbent assay, SDS-PAGE, and Western blot, respectively.

### 4.11. Western Blot

The total proteins were extracted from *T. flavidus* using RIPA lysis buffer (Solarbio, Beijing, China) and their concentration was determined using the BCA Protein Assay Kit (Pierce™, Rockford, IL, USA). Protein samples were separated using 12% SDS-PAGE and transferred onto polyvinylidene fluoride membranes. The membrane was blocked for 3 h at 37 °C with PBS supplemented with 0.5% Tween-20 and 5% skim milk, and then incubated for 1.5 h at 37 °C with anti-rTfVWD polyclonal antibody (1:500). β-actin antibody (Proteintech, Rosemont, IL, USA) was used as an internal reference. After washing four times with PBS containing Tween-20, the membranes were incubated with horseradish peroxidase-conjugated goat anti-mouse secondary antibodies (1:10,000 dilution) at 37 °C for 40 min. After washing, chemiluminescence substrates (ECL, Millipore, Billerica, MA, USA) were used to reveal positive bands, which were visualized following exposure to Hyperfilm ECL.

### 4.12. Statistical Analysis

One-way analysis of variance (ANOVA) followed by Tukey’s test was performed to compare the differences in the relevant data using SPSS software (version 24.0) (IBM, Armonk, NY, USA). Significant differences between different tissues (*p* < 0.05) were calculated and denoted by letters (a, b, c, and d); the same letters indicate no significant difference between groups. Different letters indicate statistically significant differences between groups (*p* < 0.05). All data in graphs are expressed as the means ± standard errors.

## 5. Conclusions

To the best of our knowledge, this study is the first to report the heterologous expression of TfVWD cloned in the pET-28a vector and its TTX binding ability. Molecular docking revealed that amino acids Val^115^, ASP^116^, Val^117^, and Lys^122^ are critical for TfVWD protein binding to TTX. SPR analysis showed a K_D_ for the recombinant protein and TTX binding of 2.9 × 10^−3^ M, whereas the VWD mutant did not bind TTX. TTX administration to mice via gavage demonstrated that rTfVWD exerts a neutralizing effect on toxicity. In summary, we demonstrated the active function of a new TTX-binding protein, namely, the VWD, in *T. flavidus* and identified the active sites. These data provide a theoretical basis for further exploring the molecular mechanism of TTX enrichment and transfer in pufferfish.

## Data Availability

The cDNA of the vitellogenin gene and TfVWD sequences are available from GenBank under the accession number MW603776 and MT160192.

## Supplementary Materials

The following are available online at https://www.mdpi.com/1660-3397/19/4/181/s1, Figure S1: The cDNA fragment of the vitellogenin gene; Figure S2: Construction of the recombinant expression vector.
