# Supplementary material for: Molecular Characterization of the Von Willebrand Factor Type D Domain of Vitellogenin from Takifugu flavidus
Source: Mar Drugs. 2021 Mar 25;19(4):181. doi: 10.3390/md19040181 (PMC8065724; doi:10.3390/md19040181)
Supplement: Supplementary file 1 [file marinedrugs-19-00181-s001.pdf]

GGCCACAACCCATCAGCCATGAGAGTGGTTGTGCTCGCTCTGACTCTGGCCCTCGTGGCTGGGCAGTCTCATCATCTCACACACGTGTTT 90
GAGGCTCACAAGACCTATGTCTACCAGTATGAGACGACGCTCATGGGCGGAATGCAGGAGGAGAAATTGGCAAAATCCGGACTCAACTTC 180
AGTTCTAAAGTCCTAATTAGTGCTGAGTCTGGCAACATATACCTGCTTAAGCTAGAGGAACCTGAGATCTTCGAGTTAAATGGGGTCTTG 270
TCAAAAGATCCATTGGTCCCAGCCTCCAGACTGACCGCAGCCCTGAAACATCAGTTAACGGATCCCATCAAGTTTGAGTACACCAACGGC 360
GTTGTTGGGAAGATGATGGCTCCCGCTAGTGTGTCCACACTTGTGTTGAACATTTACAGGGGTATCCTGAATATCCTTCAACTCAACATC 450
AAGAAGACGCACAACGTCTATGAGTTGCAGGAGGCTGGAGCTCAGGGAGTGTGCAAGACCACATATGCCATCTTTGAGGAGGAGAAGGCT 540
GAACAAATTCATGTGACCAAGACCAGGGATTTGAACCAGTGTCAGGAGGGATTCATCAAGGATATGGGGTTGGCATACACTGAGAGATGT 630
GTCAAGTGCCAGCAGGACACGAAGAGCCTGAGAGGATCAACAGCATACGATTACATCTTTAAGTCACACGCTGAAAGTGTCCAGATCCTG 720
CAGGTGAAGGCCAACGAGGTGATCCGGCTTGCCCCTTTGAGCGAGAGGAACGGAGCCGTTGAGATGCAGACCAAGCAAGTCTTTTCCTTC 810
GTTGAGATCCAGAAGACTCCAATCGTTGTCGGCAGCTCTCCCTACGTCGATAGTGGGTCTCTTAGGTACGAGTTTCCCAGGGAGCTTTTC 900
CAGACACCTCTGCAGCTTGTCAAGATCGTCGAATTACAGAAGCAGATTGGGGAACTTCTTGATCACCTAATCGCCAACAATGTGGAGAGA 990
GTCCGTGAGAACGCACCTGAGAAGTTTTTGGAACTGATCCAGCTTCTTCGTTCAGCCAGCGTTCAGGACCTGGAATCAATCTGGACACGG 1080
AGCAGCAAGAACACCTACAGATGGTGGGTTTGGGATTCCGTAGCAGCTACAGGAACTCCTGCTGCTCTGAGATTCATTAAAGACAAGTTC 1170
CTGAAAAAGGAAGTGTCAGTCGCTGAAATGGCCCAAGCTATGGTGACATCTGTTCACATGGTGACAGCAACCACTGAGACCATTGAGATC 1260
TTTAAGGACCTGGCAAAACGCAAGGAAATAGTGGACAGCCCATTTCTGCGTGAAATAGTTCTGCTCGGCTATGGGACCATGATTGGAAAA 1350
CAGTGTTCCCAACAAGCTGTCTGTCCTGTGGAGTCCATCCAGTTCATCCACAACCTTCTGTCTGACGCCCATTCAACCAAAAAAATCAGT 1440
GAAATCATCCTGCACCTGAAGGTTCTGGGAAACGCTGGCCTTCCTGACAGCATCAAGCCGATCACAAAGATCCTGCCCATAGCCCACTCG 1530
CCCACGGATACATCTCTGCCCATTACAGTCTATGCTGAAGCCATCATGGCCCTGAGGAACATTGCAAAGAAGGAGCCCAAACGGATCCAG 1620
GAACTGGCTCTTCAGCTGTACATGGACAAGAACCTTGACCCCGAGCTTCGTATGCTTTCCTGCATCGTCCTGTTTGAAACCAAACCTTCT 1710
CTTGGTCTGGTGACAGCACTTGCCAACATTGTGAGGACAGAGGAGAGTCTGCAGTTAGTGAGCTTTACCTACTCTCACATGAAGTCCCTG 1800
AGCAGGAGCACCTCCATCGTCCACTCATCAGTTGCTGCAGCTTGCAACATTGCTCTCAGAATGTTGAGCCCAAAGTTGAACAGACTGAGC 1890
CTCCGTTTCAGCAGAGCCATCCATCTGGATGTCTATAACAGTCATTACATGCTCGGCGCTGCGGCCAATGCTTTCTACATCAACAATGCT 1980
GCCAGCATTTTGCCCAGAAATTTCATGGCGAAGGCCAGTGCATATGTTTCTGGAGCTGCTGTTGATGTTCTGGAGGTTGGAATGAGAACT 2070
GAAGGACTGCAGGAGTTACTTCTGAAAAACCCCACAATTCTCAACAACGAGGACAGGATCACCAAGATGAGGAAAGCCATCCAAGCTCTT 2160
TCTCATTGGAAGAGTTCACCCAATGCAAAACCTCTGGCTTCTTTCTATGTCAAGTTCATGGGGCAAGAAATTGCCTTTGCCAGCATTGAT 2250
CAAGCACTTCAGCTTCCTAGTGTTGAATCTATGAGGACCATTGGTAATGACATCATCAGAGAAATGGCATCTGGCATCTCGGTAAACATC 2340
GTCAAGCCTCTGCTGGTCAGTGAGGTGAGGCGTATCATGCCCACTGCTGCTGGTTTTCCATTGGAGTTCAGTCTGTTAACAGCTGCCGTG 2430
ACAGCTACAGGTGTCAGAGTCAAGGCCACCATGACACCAGCCCTGCCGGAAAACTTCAAACTCATTGAACTCCTGCAGACCGACATGCAA 2520
ATCGAGAGTGAGATCAAACCAAGCATTGCTGTGAACACCCCTTGCAACCATGGGAATAAACACTGCTATGTTCCAGTCGGGAATTGTCTCC 2610
AGAGCTAAACTCACCACCACTGTCCCCACCAACATTGTTGCAAGGCTAAACCTCCACAAGGGCTACTTTAAGATTGAAGCTCTGCCCGTT 2700
TCCTTGCCTGAGAACATTGCAGCCATGCACATTGAAACCCTAGCTGTGACCAGAAACATCGAGGACTTAAGTGCTGCAAGACTAACCCCG 2790
ATCATCCCGGAACAAGTCCAGAATCTTGCTTCAATGGAGGATCTTACATCCCGGAATGAAAAAAGCCAGTCACATTCGTCAGAGGTTTAT 2880
TACCAAGACCATCCACGGCCATTCACAAAGTCTAAAGTTGCCCGGGTTAAGAATTACAGCGCTGAAATCCTGGGACTGAAGGGCAGTGTG 2970
ACTATGGTCACTCACAATGCTGCTTTCATCATGGACGTTCCCCTGTACAGACTAGCTGGAACACACTATGCTGCTGTTTCTATCACACCA 3060
GTTCCAGGAGAAACATATGACAGAGTGGAGCTGGAAGTTCAAGTCGGACCGCAGGCAGAAGAGATGCTGATGAAAAAGATCAATCTGAAC 3150
GATGAGGAAATAACTGAGGGGAAACCAGTCCTGATAAAGCTCCAACGGCTACTGAAGCCTACTGTCAACAGTGCATCATCAGTGAGTGGA 3240
AAAAGCAAGGGCAGCAGCAGGGGCAGTGACTCCAGATCTGTTATAAACTTATCCAGTGGCAGCACCTCTGCATCGAACACCACGTCACGT 3330
TCATCACGACAGAGGAAACATGTCACGAAGCGAATTAAAACCCATAAGTTCAACAAGAACCACAGGAAGCAGTATAAAAGTCAAATGGCA 3420
GATTCCTCTGGCAGTGCTGCAAGCTTTGAGGCAGTCTACGAGCAGAACAAATTCCTTGGCAGCTCTGGAGTTCCTAGCTTTGCTGTAGTC 3510
GTGCGTGCTGTCAAAGGTAAGATGGTAGCAGGATACCAGTTGGCAGTCTACATGGACAAACCCACGAATAGAATTCAGATGATTCTGGCT 3600
GATCTGGACTCTGAAAACAACTGGAAACTCTGTGCTGATGGAATTGTGCTTAGCGAGCACAGAGTCGTAGCTAGAATTGGCTACGGAGCT 3690
GCATGCAACAAGTACAGGGCCACCATCACTGCAGAGACTGGTCTCGTTGGTCCGAGCCCTGCAGGTCGCCTCAGAGTGGACTGGAACGAA 3780
ATACCCGATGCCCTTAAGCGCACGTTGGAAAAGGCAATGAAGAGCATTCCTTTATCCATCGAGTGTACTTTTGACAAGCAAAGGAGCAAA 3870
AACACCGCAAACCAGGTCTCAGTTATCATAGTTGCTCCGACCGAGAAGACCCTCGACATCATTCTCAAAACACCAACGTATACCTTCTTC 3960
AACCTGGATGTGCCTCTTCCGATTACCCTTCCAATCAATGAAATACATGGCCTCGCCCCCTTTGGTGACATCGTTGATGAACTCCATGTT 4050

TfVWDF primer →

GTGGCAGCCAAGGCCATAGCAGCTCAGTGTAA**CTACGCCCAGAACTTGCTGAC**CACCTTCAACGACATCAAATACGAGCCCCAGATGCCG 4140

T T F N D I K Y E P Q M P

CCGTCTTGCTACCAAATTCTGGTTCAGGACTGCACACCCGAGCTGAAATTCATTGTTATGCTGAAGAATGATAACTTTGAACAGAAACAC 4230

P S C Y Q I L V Q D C T P E L K F I V M L K N D N F E Q K H

ATAAATATAAAGATCGCTGACATCGACATCGACCTCTTCCCCAAGAGTGGCAACATAGGTGTGAAGGTCAATGGTGTGGAAATACCAATG 4320
I N I K I A D I D I D L F P K S G N I G V K V N G V E I P M
GAAAACCTGCCATACCATCATCCCACAGTTAAAATCCAGATCAGGCAAAAGGGTGAAGGCATCTCTGTTGTTGCTCCCAGCCTCGGGCTT 4410
E N L P Y H H P T V K I Q I R Q K G E G I S V V A P S L G L
AGTGAAGTCTATATGGACAGTAAATCATGGAAGGTTGATGTTGTGGACTGGATGAAGGGACAGACTTGTGGACTCTGTGGGAAGGCTGAT 4500
S E V Y M D S K S W K V D V V D W M K G Q T C G L C G K A D
GGGGAGATCAAACAGGAGTTCCGTATGCCCAACGGGCACCTGACCAAGAATGCGGTCACTTACGCTCATTCCTGGATCCTGGCGGCCGAG 4590
G E I K Q E F R M P N G
AGCT**GCAGAGACAACAGCGAGTGC**CGAATCAAGCTCGACTCTGTAGAGCTGGAAAAGCAGGCGGTCATCTATGGTCAGGAATCTAGATGT 4680
TfVWDR primer
TTCTCCGTTGAGCCGGTGCTCCGCTGCCTGCCCGGCTGCTTCCCTGTGAAGACCACTGCCGTCACCGTTGGCTTCCACTGCGTGGCTGCT 4770
GATTCCAATGTGAACAAATCGGAGGTTCTCAGAGGAATCCAAGGGAAGAGGGTTGACCTGAGGGAAAAAGCTGATGCTCACCTGGCCTGC 4860
AGCTGCACGGCTCAGTGTGTTTGA 4884

Figure S1. The cDNA fragment of the vitellogenin gene. The VWD domain sequence is underlined, the primers are shown in bold, and the conserved Cys residues are shaded in gray.

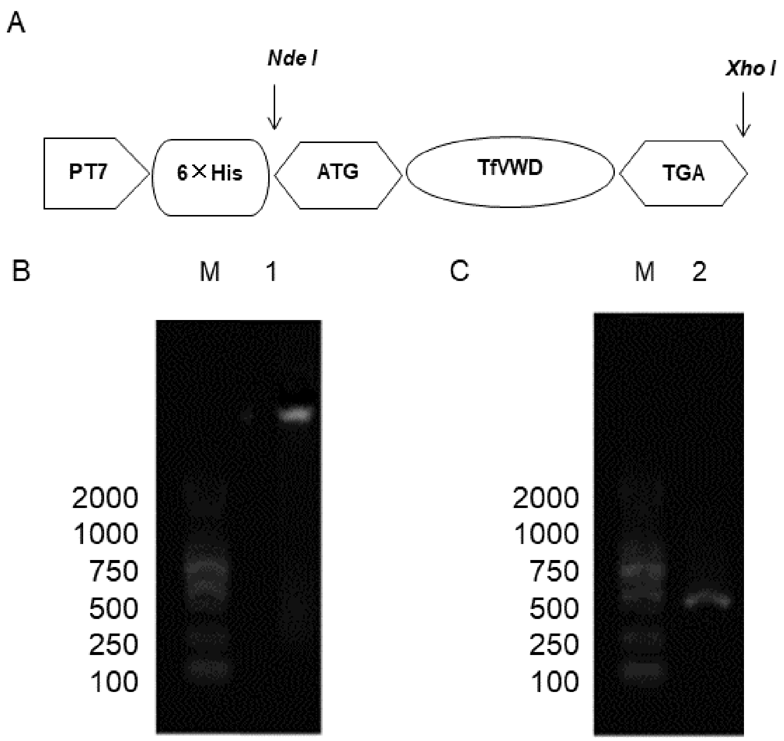

Figure S2. Construction of the recombinant expression vector. A. Structural map of the pET28a/TfVWD recombinant plasmid vector. B. Electropherogram of the pET28a plasmid following double enzymatic digestion. Lane M: DL2000 Plus DNA Marker; lane 1: Following enzymatic cleavage of the vector. **C** Electropherogram of the PCR product of the TfVWD expression fragment. Lane M: DL2000 Plus DNA Marker; lane 2: PCR product of TfVWD.
